# Assessing critical gaps in COVID-19 testing capacity: the case of delayed results in Ecuador

**DOI:** 10.1186/s12889-021-10715-x

**Published:** 2021-04-01

**Authors:** Irene Torres, Rachel Sippy, Fernando Sacoto

**Affiliations:** 1 Fundacion Octaedro, El Zurriago E8-28, Quito, Ecuador; 2 Institute for Global Health & Translational Sciences, State University of New York Upstate Medical University, Syracuse, New York USA; 3 Ecuadorian Society of Public Health, Quito, Ecuador

**Keywords:** Covid-19, Testing, Health policy, Inequality

## Abstract

**Background:**

Testing is crucial for COVID-19 response and management, however, WHO’s preparedness index omits estimations of actual testing capabilities, which influence the ability to contain, mitigate and clinically manage infectious diseases. With one of the highest excess death rates globally, Ecuador had a comparatively low number of confirmed COVID-19 cases, which may have been influenced by limited availability of data for decision-making due to low laboratory capacity.

**Methods:**

We examine de-identified data on 55,063 individuals with suspected COVID-19 between February 27 and April 30, 2020 included in the RT-PCR testing database collected by the Ministry of Health. Processing times and rates per province, and the number of pending tests, were tallied cumulatively. We assessed the relationship between sample shipping, laboratory capacity and case completion using a negative binomial generalized linear model.

**Results:**

The national average time for case completion was 3 days; 12.1% of samples took ≥10 days to complete; the national average daily backlog was 29.1 tests per 100,000 people. Only 8 out of 24 provinces had authorized COVID-19 processing laboratories but not all processed samples. There was an association between samples coming from outside the processing laboratory province, the number of other samples present at the laboratory during processing, and the amount of time needed to process a sample. Samples from another province took 1.29 times as long to process, on average. The percentage of pending results on April 30 was 67.1%.

**Conclusion:**

A centralized RT-PCR testing system contributes to critical delays in processing, which may mask a case burden higher than reported, impeding timely awareness, and adequate clinical care and vaccination strategies and subsequent monitoring. Although Ecuador adapted or authorized existing facilities to address limitations in laboratory capacity for COVID-19, this study highlights the need to estimate and augment laboratory capabilities for improved decision making and policies on diagnostic guidelines and availability. Support is needed to procure the necessary human and physical resources at all phases of diagnostic testing, including transportation of samples and supplies, and information management. Strengthening emergency preparedness enables a clear understanding of COVID-19 disparities within and across the country.

**Supplementary Information:**

The online version contains supplementary material available at 10.1186/s12889-021-10715-x.

## Background

WHO has emphasized the importance of testing during the coronavirus disease 2019 (COVID-19) pandemic [1] and, for this, to expand the capacity of national laboratories [2]; however, actual testing capabilities are often not analyzed when rolling out country diagnostic strategies. Even among highly-prepared health systems, diagnostic capacity and public health reporting systems have been stretched to the limit with rapid local transmission of severe acute respiratory syndrome coronavirus 2 (SARS-CoV-2) [3], which causes COVID-19. Country self-assessment for WHO’s preparedness index is subjective and omits expressly requiring to estimate laboratory capacity according to the time it takes from reporting a case, and taking a sample, to processing it and notifying results, depending on location [2]. This poses a risk during an “unusual health event” [4] such as the current pandemic and in its aftermath, since testing will continue to be crucial for healthcare decision-making in the COVID-19 response [5], to contain and mitigate, as well as manage, the disease.

Assessing coronavirus testing competence seems to have been sidelined by modeling of transmission rates and impact of non-pharmaceutical measures, and estimations of health system overburden. Shortage of testing supplies in many countries, coupled with high demand for testing in strained public health systems has caused a backlog of patient samples awaiting results and delays in reporting [5], and, therefore, may have impeded adequate tracing. To the best of our knowledge, while there are no studies on delays in RT-PCR results in other countries in the region, the media reported backlogs in countries that were severely impacted by the pandemic, such as Brazil [6], Chile [7], Colombia [8] and the United States [9].

Ecuador has reported some of the highest mortality and excess mortality rates due to COVID-19 [10] and experienced the unexpected collapse of public health services as the epidemic accelerated across the country. Compared to the burden of COVID-19 in another middle-income South American country such as Uruguay (173 deaths pmp [11]), Ecuador had a high mortality rate (882 pmp) [11] by February 26, 2021. The country’s positivity rate (28% in 56,000 tests pmp) is higher than Uruguay (5.7% in 290,000 tests pmp), the United States (8% in 1 million tests pmp), Chile (8.8% in in 490,000 tests pmp) and Colombia (19.6% in 223,000 tests pmp). In Ecuador, information on COVID-19 has been highly centralized by the national government [12]. The disparities of burden observed between this and other countries may have been influenced by delayed or limited availability of data due to low laboratory capacity, which, combined with centralized control of information, limited decision-making.

Similarly to many countries in low-income and middle-income countries (LMICs) facing COVID-19, Ecuador does not produce laboratory supplies, and disruption of global transportation diminished imports while the national lockdown delayed customs processing. There have been shortages of reagents, RNA extraction kits and swabs, and prices of the essential personal protective equipment required to collect and process samples such as N95 masks have increased, resulting in more expensive testing. In addition, not all laboratories have the capability to process large numbers of samples because of gaps in the workforce, including technicians and administrative assistants, as well as low availability of laboratory equipment and computing resources to process data and aid in workflow management. This is why Ecuador may be under-testing its population, with only 46,209 tests pmp, compared to 216,382 pmp in Uruguay as of January 18, 2021 [11].

Agreements permit patient referrals and requests for medical tests across the Ministry of Public Health (MoH), the Ecuadorian Institute of Social Security and the private sector, but inter-institutional coordination is inefficient. Moreover, persistent social, geographic and economic inequalities in Ecuador negatively influence access to health care [13], with health services not being similarly available across different municipalities and provinces, and urban areas having higher concentrations of health providers. Further, Ecuador’s ongoing economic crisis has prompted underfunding of the MoH while patterns in laboratory diagnostics indicate possible shortcomings in capabilities [14]. As a reference, more remote provinces such as Bolívar, Zamora Chinchipe and Galápagos do not have healthcare resources such as intensive care units (ICU), and Cotopaxi, Cañar and Morona Santiago have ICU bed rates as low as 0.11, 014 and 0.15 per 10,000 people, respectively [15]. In this context, access to health services may involve transportation across provinces for hundreds of kilometers [16].

As transmission continues, testing limitations of the country [4] have yet to be assessed and correspondingly addressed. In this study, we present the first systematic assessment of COVID-19 diagnostic capabilities in Ecuador, a country of 17.6 million, providing a model for estimating delays due to laboratory capacity, patient access to care or the lack of widespread processing laboratories that may show disparities in testing availability. The estimation method may be used by Ecuador and comparable middle-income countries to improve decision making and policies on national diagnostic guidelines and availability for COVID-19 and other emerging infectious diseases.

## Methods

Data on individuals with suspected SARS-CoV-2 infection were included in the national RT-PCR testing database collected by Ecuador’s MoH. Duplicate tests per individual were not part of the database. De-identified data covering case notification dates of February 27—April 30, 2020 were provided by MoH for the present study. No data were excluded from the study unless otherwise indicated. At the time of analysis, individuals had a case designation of “confirmed”/“discarded” based on diagnostic testing results, or “probable”/“suspected” based on epidemiological evidence of exposure to another COVID-19 case or reporting of symptoms. Sample availability, type, and processing laboratory were also available. The data included dates for initial symptoms, healthcare attention, sample collection, and result notification. Province-level case rates were calculated using all confirmed, probable, and suspected cases by the patient’s recorded province of residence and the projected province population for 2020 [17]. To calculate sample collection, positive, negative, processed and pending case rates, the number of individuals with samples were tallied cumulatively by sample collection date and recorded province of residence. For those with no sample collection date, the date of healthcare attention was used for the sample collection date.

Notification dates were used to determine the date of test result. Individuals with a notification date occurring before the date of healthcare attention were excluded. The number of processed tests (i.e. test with laboratory results of positive, negative, inconclusive, or not processed) were also tallied cumulatively by notification date and recorded province of residence. Pending tests were the daily number of results remaining after subtracting daily processed tests from daily collected samples. To calculate mean province processing times and rates, only dates during which a province had samples were included. To assess the relationship between sample shipping, laboratory capacity, and case completion, data with complete laboratory information were analyzed. Individuals with a sample collection date after the notification date were excluded. Based on the origin of the sample and location of the laboratory, it was determined if the sample came from within the same province as the processing laboratory or if it had been shipped from another province. In addition, the average number of samples present the laboratory during the processing of each sample was calculated. The relationship between these variables and the number of days for processing (days from sample collection to case notification) was assessed using a negative binomial generalized linear model. Information on authorized laboratories were provided by the MoH and the Agency for Quality Assurance in Health Services and Prepaid Medicine (ACESS for its Spanish acronym).

Data were processed, analyzed, and visualized using R version 3.4.1 [18] in RStudio [19] with packages maptools, rgeos, raster, rgdal, maps, MASS and RColorBrewer.

## Results

**Fig. 1:**
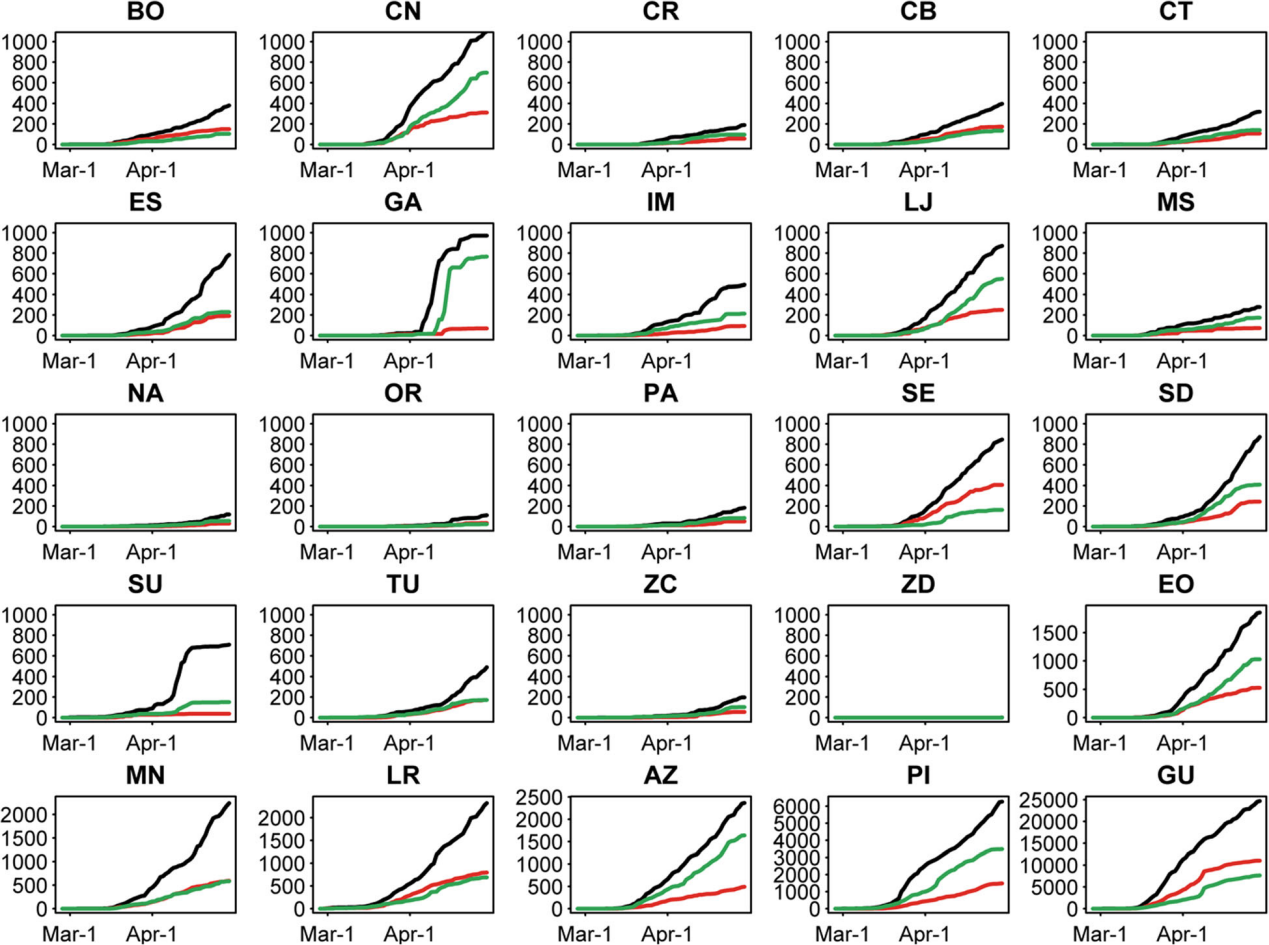
Total Samples Collected and RT-PCR Test Results by Date in Each Province. For each province, the cumulative number of samples taken and test results are given. Total collected samples are in black, positive samples are in red and negative samples are in green. AZ = Azuay, BO=Bolívar, CN=Cañar, CR = Carchi, CB=Chimborazo, CT = Cotopaxi, EO = El Oro, ES = Esmeraldas, GA = Galápagos, GU = Guayas, IM = Imbabura, LJ = Loja, LR = Los Ríos, MN = Manabí, MS = Morona Santiago, NA = Napo, OR = Orellana, PA = Pastaza, PI=Pichincha, SE = Santa Elena, SD=Santo Domingo de los Tsáchilas, SU=Sucumbíos, TU = Tungurahua, ZC = Zamora Chinchipe, ZD = Zona No Delimitada

Between February 24th and April 30th, there were 55,063 individuals with probable COVID-19 in the MoH database. Samples were collected from 49,146 (89.3%) individuals; 98% of samples were naso- or oro-pharyngeal swabs, 1% were sputum, and 1% were another method. Total samples collected and reported by day in each province are in Fig. 1. For many provinces, the number of processed tests with results (positive/negative) rises steadily with the number of samples collected (e.g. Chimborazo, Azuay), illustrating that sample processing matched the collection rate. For other provinces, test result counts reach a plateau (e.g. Guayas), indicating that sample processing could not keep up with the collection rate.

Between February 27th and April 30th, 2020, 17,806 people in Ecuador were positively diagnosed, with an additional 16,389 suspected cases and 1613 probable cases. For this duration, the national case rate was 204.5 cases per 100,000 people. Galápagos Province (islands located 600 miles offshore) had the highest rate of cases, with 789.9 positives per 100,000 people (261 cases total). The next highest rates were in the coastal provinces of Guayas (455.5 cases per 100,000, 19,983 cases total) and neighboring Santa Elena (256.5 cases per 100,000, 1029 cases total). Case rates for all provinces are in Supplementary Figure 1.

**Fig. 2:**
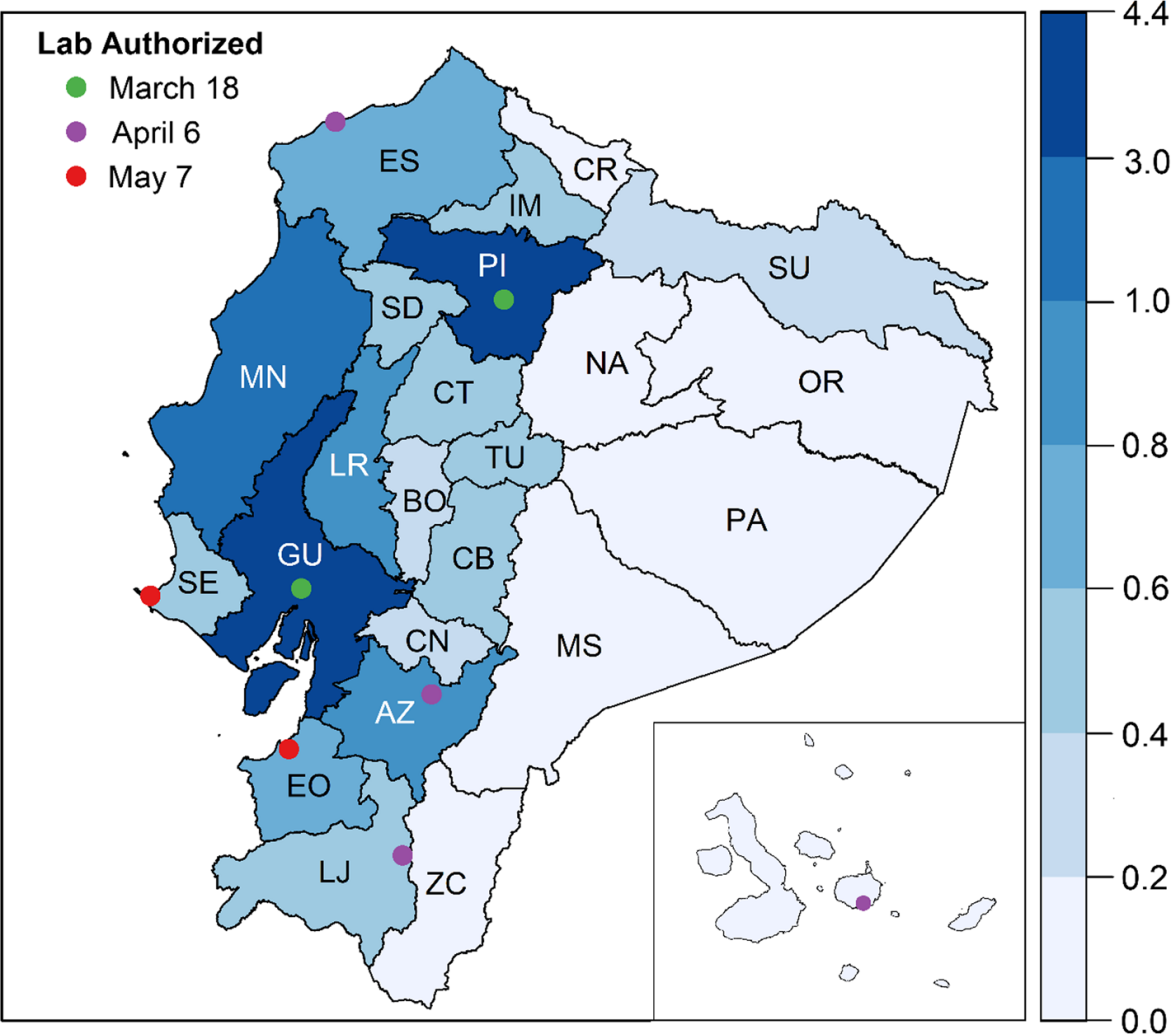
Location of Laboratories Authorized to Process COVID-19 Samples and Population Distribution by Province. For each province, the 2020 population (in millions) is indicated by color. The location of laboratories authorized for the processing of COVID-19 samples is given by points on the map, with colors to indicate the date by which these locations were authorized (green = March 18th or sooner, purple = April 6th, red = May 7th). The Galápagos Islands are inset. AZ = Azuay, BO=Bolívar, CN=Cañar, CR = Carchi, CB=Chimborazo, CT = Cotopaxi, EO = El Oro, ES = Esmeraldas, GU = Guayas, IM = Imbabura, LJ = Loja, LR = Los Ríos, MN = Manabí, MS = Morona Santiago, NA = Napo, OR = Orellana, PA = Pastaza, PI=Pichincha, SE = Santa Elena, SD=Santo Domingo de los Tsáchilas, SU=Sucumbíos, TU = Tungurahua, ZC = Zamora Chinchipe

Laboratories require specific authorization from ACESS to process SARS-CoV-2 samples and testing capabilities extended slowly in the country. Diagnostics began in two public laboratories, first in the large coastal city of Guayaquil, the country’s hotspot, and then in Andean highland city of Quito, the capital city; on March 18, two private facilities became authorized to process samples in Quito. On April 6, apart from two universities conducting limited research, additional laboratories were authorized in the cities of Guayaquil (2 public, 3 private), Cuenca (3 public, 2 public), Quito (1 public, 2 private), Esmeraldas (1 public) and Loja (1 private) and the Galápagos Islands (1 private), for a total of 19 laboratories in 6 municipalities. Laboratories in two additional coastal cities (Machala and La Libertad) were added on May 7, for a total of 8 municipalities (out of 221) in 8 (out of 24) provinces with processing capabilities. Laboratory locations are indicated in Fig. 2, with province-level population for context.

**Fig. 3:**
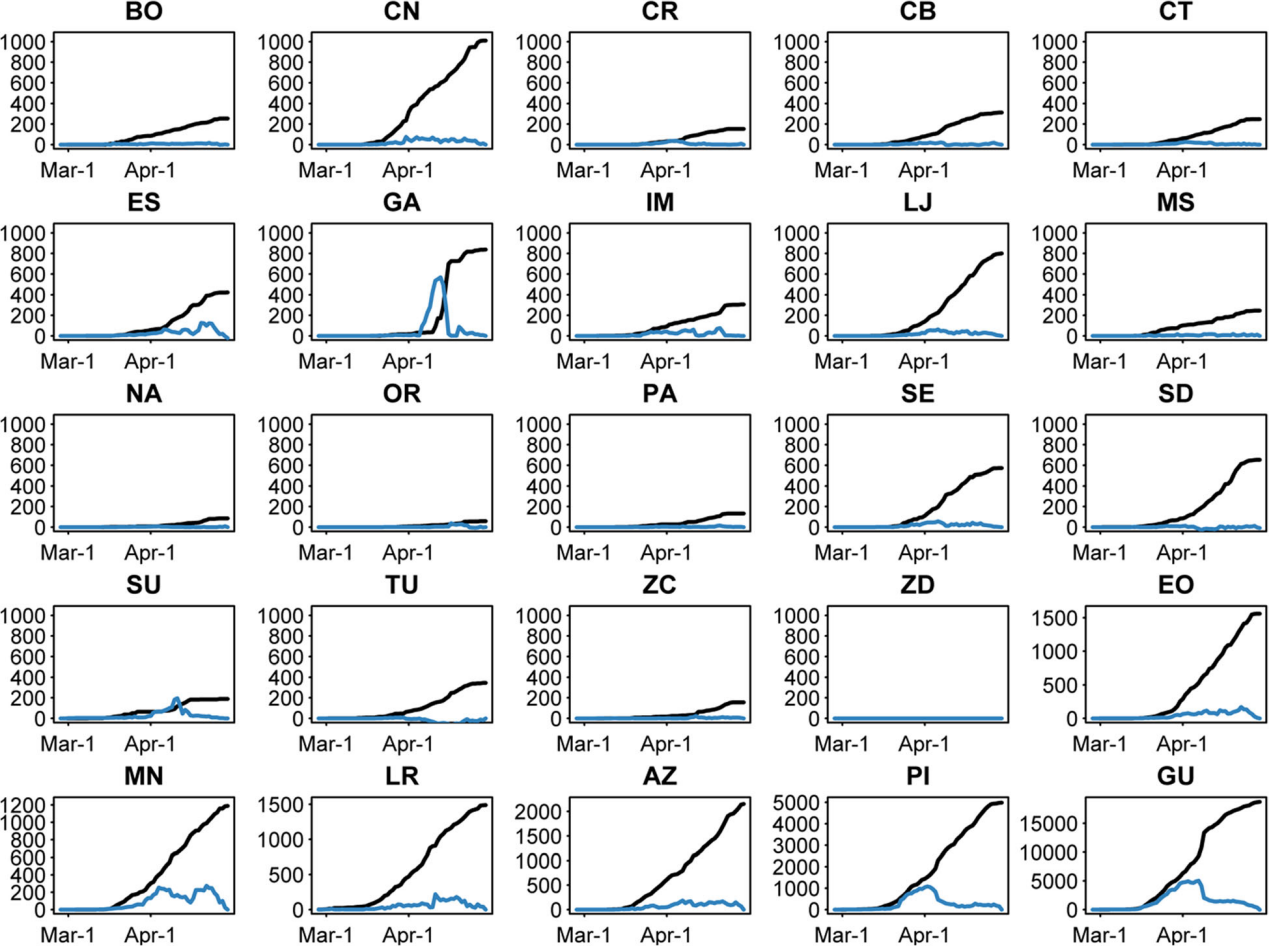
Processed and Pending RT-PCR Tests by Date in Each Province. For each province, the cumulative number of processed tests are given in black, and the cumulative number of pending results are in blue. AZ = Azuay, BO=Bolívar, CN=Cañar, CR = Carchi, CB=Chimborazo, CT = Cotopaxi, EO = El Oro, ES = Esmeraldas, GA = Galápagos, GU = Guayas, IM = Imbabura, LJ = Loja, LR = Los Ríos, MN = Manabí, MS = Morona Santiago, NA = Napo, OR = Orellana, PA = Pastaza, PI=Pichincha, SE = Santa Elena, SD=Santo Domingo de los Tsáchilas, SU=Sucumbíos, TU = Tungurahua, ZC = Zamora Chinchipe, ZD = Zona No Delimitada

During the study period, Ecuador processed 36,927 tests (75.1% of 49,146 samples). There were 49,118 samples included in the analyses of test completion and backlog. On average, Ecuador completed 6.2 tests daily per 100,000 people. Provinces with the lowest test completion rates (Fig. 3), between 1.2 and 1.3 tests per 100,000 per day on average, are in the highlands (Cotopaxi, Chimborazo and Tungurahua). Provinces with the highest completion rates (Fig. 3) are Galápagos (88.4 tests per 100,000 per day on average), Cañar (7.6 tests per 100,000 per day on average) and Guayas (7.0 tests per 100,000 per day on average). Although completion rates varied by province, they also varied over time during the pandemic. Galápagos had a high completion rate but a comparison of the processed and pending results (Fig. 2) shows that Galápagos had a large backlog of tests during the month of April. This pattern can also be seen in the data from Sucumbíos.

**Fig. 4:**
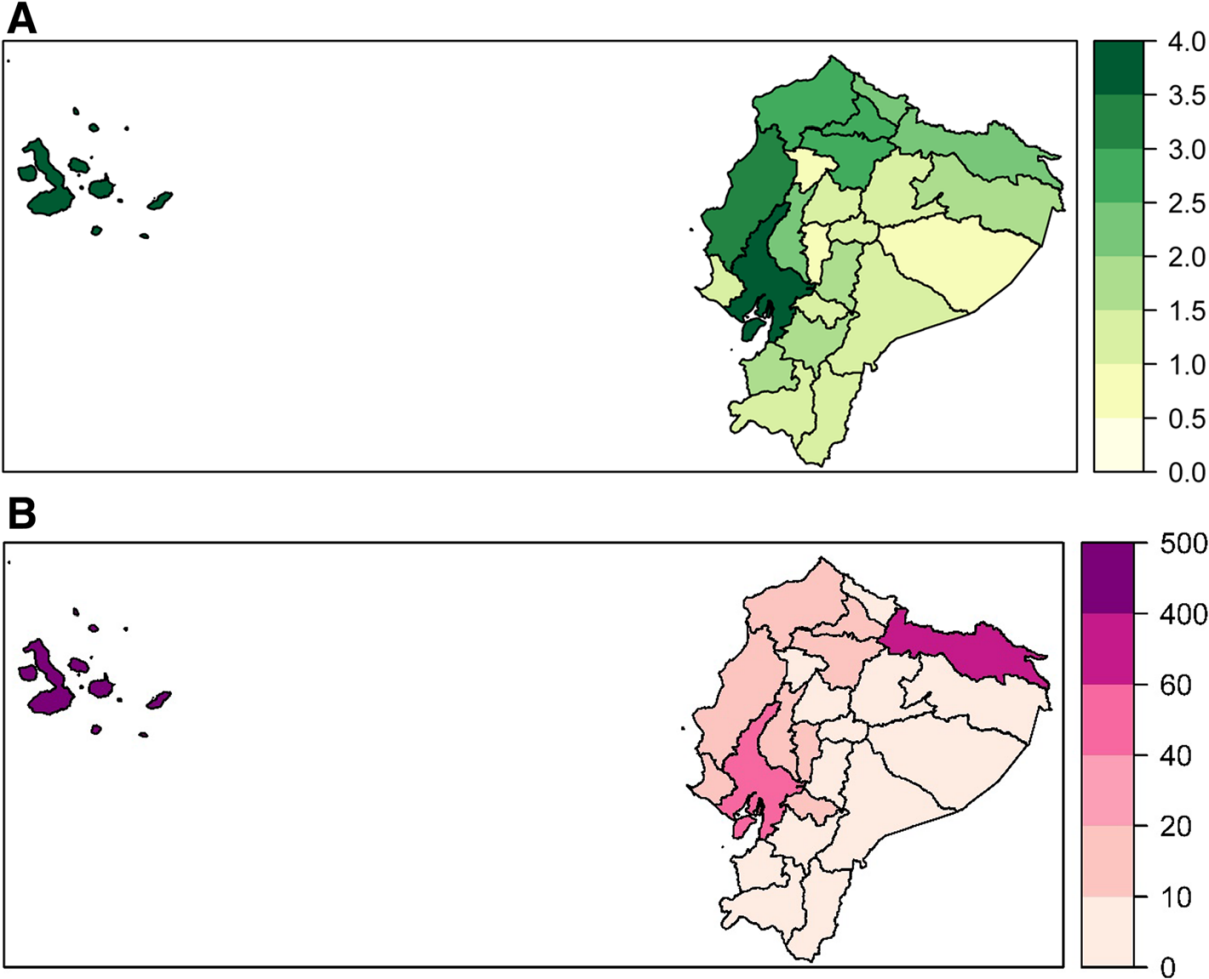
Case Notification and RT-PCR Test Completion by Province. For each province, the mean time from attention to notification by province is given, with more intense green indicating a longer mean processing time (**a**). For each province, the mean test pending rate per 100,000 province inhabitants per day is given, with more intense purple indicating a higher pending test rate (**b**)

The national average time for test completion (from healthcare attention to notification) is 3.0 days (range: 0—60). Nationally, 12.1% of samples took 10 days or more to complete. The provinces with the longest mean test completion times (Fig. 4a and Supplementary Figure 2) are Guayas (3.8 days, range: 0—39 days, 17.5% 10 days or longer), Galápagos (3.7 days, range: 0—17 days, 1.8% 10 days or longer) and Manabí (3.4 days, range: 0—35 days, 11.8% 10 days or longer).

During the study period, Ecuador had an average daily backlog of 29.1 tests per 100,000 people (12,216 tests pending). The highest mean daily backlog of tests (Fig. 4b) was in Galápagos (404.1 pending tests daily per 100,000, 132 total pending), Sucumbíos (76.7 pending tests daily per 100,000, 519 total pending) and Guayas (57.7 pending tests daily per 100,000, 5944 total pending).

Among samples with complete laboratory information meeting inclusion criteria (*n* = 32,166), there was an association between samples coming from outside of the processing laboratory province (i.e. those samples that had been shipped), the number of other samples present at the laboratory during processing, and the amount of time needed to process a sample. Compared to samples taken in the same province as the processing laboratory, samples from another province took 1.29 times as long to process, on average (risk ratio: 1.29, 95% confidence interval: 1.24—1.33, *p* < 0.001). For every 100 additional samples present at the processing laboratory, it took 1.07 times as long to process the sample, on average (risk ratio: 1.07, 95% confidence interval: 1.06—1.07, p < 0.001).

Following poor information disclosure on cases at the beginning of the pandemic [20], irregular increases and decreases in daily reports of the number of SARS-CoV-2 tests and confirmed cases have given indications of barriers in testing [21]. The proportion of pending results did not change substantially, at 19.3% on May 28, compared to 24.9% on April 30. In addition, the lags between confirmed cases and deaths notified the MoH and the National Risk and Emergency Management Service (SNGR for its Spanish acronym) are evidence of additional lags between the central MoH database and reporting to the public.

## Discussion

The results of this study highlight the critical need to improve decision making and policies on national diagnostic guidelines and availability for COVID-19 and other emerging infectious diseases, by adequately estimating and augmenting laboratory capabilities. Ecuador maintains a centralized RT-PCR testing system wherein each province’s samples are sent to a limited number of regionally located laboratories for processing. Our analysis shows that this system has contributed to delays in processing. Testing burden at each laboratory also influences processing delays, but the larger impact on processing time is whether the sample came from another province, i.e., had to be shipped from another province. Furthermore, laboratories must send the results to the originating laboratory before formal notification to the MoH central database, which influences testing completion and produces an additional delay in reporting of results.

An appropriate public health response demands strong laboratory capacity, articulated with an efficient epidemiological surveillance and adequate clinical care. Ecuador struggled to maintain a sample processing pace consistent with the increase in COVID-19 cases in the city of Guayaquil, reflecting the strain to public health services during intense outbreak situations. The large coastal city of Guayaquil, located in Guayas province and the epicenter of the COVID-19 outbreak for the study period, had a high burden of cases. Guayas province was able to maintain a high test completion rate during the study period but also had the longest mean case completion time, a high pending test rate, and the highest number of pending tests. Delays may have been due to samples shipped from neighboring Manabí and Los Ríos provinces, which had high case rates but no RT-PCR processing laboratories.

The Galápagos Islands were also heavily impacted, with the highest case rate and the highest mean daily backlog of tests. In Galápagos, there were no processing laboratories on the islands until April 6, meaning all samples had to be shipped to mainland Ecuador for processing during the national lockdown, which contributed to the delay in their case completion. Provinces in the central highlands of Ecuador (Cotopaxi, Chimborazo, and Tungurahua) and in the rainforest (Sucumbíos, at 265 km from the nearest processing laboratory in Pichincha [16]) also lacked diagnostic laboratories during the study period, which likely explains their low test completion rates.

A high case rate signaling widespread community transmission in the Americas region implies that countries with initial limitations [6–9] were strongly affected by the pandemic, even after ramping up testing. In the case of Ecuador, the country was unable to rapidly scale up national diagnostic capacity corresponding to the magnitude of the pandemic; by May 7, only 3.6% of municipalities could process RT-PCR tests for SARS-COV-2. Lack of synchronization in public reporting increased these reporting delays, further impeding timely awareness of increases in community transmission of SARS-CoV-2, particularly when publicly shared information was limited to data at a wider scale.

## Conclusion

If SARS-CoV-2 testing in Ecuador continues to be restricted to few locations, Ecuador’s public health tracking and response efforts will be hindered, while inequalities among municipalities and provinces will persist or become exacerbated, alongside an equivalent impact of COVID-19. Although the government has appeared to improve direct laboratory notification to the MoH, it is crucial to synchronize or automate information on confirmed tests for local-level public reports, so that knowledge essential to decision making is more rapidly disseminated than at present [12]. In sites without laboratory capacity, the government should be prepared to rapidly improve sample collection and shipment in case of a localized surge in cases. Overall, both private and public laboratories will need support to procure the necessary human and physical resources at all phases of diagnostic testing, especially if there are not enough trained personnel and equipment, including for transportation of samples and supplies, and information management. All this is essential to monitor the health condition and spread of cases, calculate the local reproduction numbers, and project overburden of health services.

Continuing limitations in RT-PCR testing capacity imply that Ecuador may not be able to engage in a systematic and active contact tracing and timely detection—especially of mild and asymptomatic cases—all of which are critical for current mitigation efforts and planning quarantine and vaccination strategies. In particular, delayed results may mask a higher than reported case burden, placing underprivileged municipalities or entire provinces at higher risk. At the same time, since even effective contact tracing and testing, together with isolation, may be insufficient to controlling COVID-19 outbreaks, countries working to shorten delays in test processing and notification must plan for further interventions. Given the high rate of confirmed deaths, Ecuador has an urgent need to address limitations in laboratory capacity more creatively, for example, adapting existing facilities like it originally did in the Galápagos Islands. Notwithstanding, laboratory authorization for processing and import of testing supplies need attention and support from the government. Finally, strengthening emergency preparedness and routine epidemiological surveillance for effective prevention and post-vaccination monitoring permits further investigation and understanding of COVID-19 disparities within and across provinces.

## Supplementary Information

**Additional file 1.**

## Data Availability

Data is available upon request from the Ministry of Health.
