# Supplementary material for: Assessing critical gaps in COVID-19 testing capacity: the case of delayed results in Ecuador
Source: BMC Public Health. 2021 Apr 1;21:637. doi: 10.1186/s12889-021-10715-x (PMC8013207; doi:10.1186/s12889-021-10715-x)
Supplement: Supplementary file 1 — Additional file 1. [file 12889_2021_10715_MOESM1_ESM.docx]

Supplementary Figures

# Assessing critical gaps in COVID-19 testing capacity: the case of delayed results in Ecuador

Irene Torres^1^, Rachel Sippy^2^, Fernando Sacoto^3^

^1^ Fundacion Octaedro, El Zurriago E8-28, Quito, Ecuador, [irene.torres@octaedro.edu.ec](mailto:irene.torres@octaedro.edu.ec)

^2^ Institute for Global Health & Translational Sciences, State University of New York Upstate Medical University, New York State, USA

^3^ Ecuadorian Society of Public Health, Quito, Ecuador

#
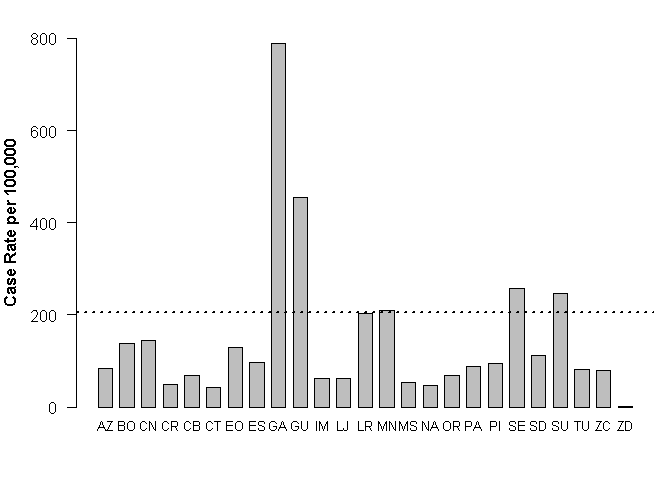


AZ=Azuay, BO=Bolivar, CN=Cañar, CR=Carchi, CB=Chimborazo, CT=Cotopaxi, EO=El Oro, ES=Esmeraldas, GA=Galápagos, GU=Guayas, IM=Imbabura, LJ=Loja, LR=Los Ríos, MN=Manabí, MS=Morona Santiago, NA=Napo, OR=Orellana, PA=Pastaza, PI=Pichincha, SE=Santa Elena, SD=Santo Domingo de los Tsáchilas, SU=Sucumbíos, TU=Tungurahua, ZC=Zamora Chinchipe, ZD=Zona No Delimitada

___________________________________________________

**Figure S1. COVID-19 rate by province.** The number of COVID-19 cases per 100,000 people are given for each province. The national rate is indicated with a dotted line.


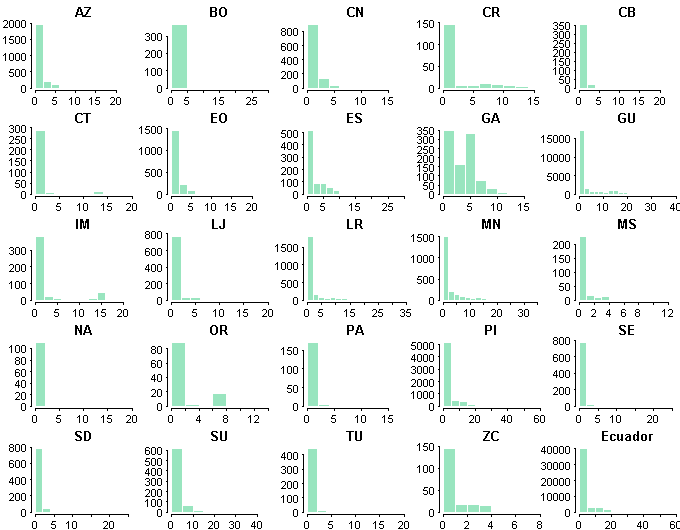


AZ=Azuay, BO=Bolivar, CN=Cañar, CR=Carchi, CB=Chimborazo, CT=Cotopaxi, EO=El Oro, ES=Esmeraldas, GA=Galápagos, GU=Guayas, IM=Imbabura, LJ=Loja, LR=Los Ríos, MN=Manabí, MS=Morona Santiago, NA=Napo, OR=Orellana, PA=Pastaza, PI=Pichincha, SE=Santa Elena, SD=Santo Domingo de los Tsáchilas, SU=Sucumbíos, TU=Tungurahua, ZC=Zamora Chinchipe, ZD=Zona No Delimitada

___________________________________________________

# Supplementary Figure 2. Time for case completion by province. For each province, the distribution of case completion times (time in days from healthcare attention to notification) for each province and nationally are given.
